# Construction of superhydrophobic graphene-based coating on steel substrate and its ultraviolet durability and corrosion resistance properties

**DOI:** 10.1038/s41598-023-27647-8

**Published:** 2023-01-11

**Authors:** M. E. Mohamed, P. S. Mekhaiel, F. M. Mahgoub

**Affiliations:** 1 Chemistry Department, Faculty of Science, Alexandria University, Alexandria, Egypt; 2 Materials Science Department, Institute of Graduate Studies & Research, Alexandria University, Alexandria, Egypt

**Keywords:** Chemistry, Engineering, Materials science

## Abstract

For the first time, a facile and environmentally friendly approach for producing high-quality graphene from the biomass of banana leaves is described in this paper. Two rough coats of Ni-graphene, Ni@G, and Ni-graphene doped with chromium, Ni@Cr-G, were created on steel substrates by electrostatic deposition. These coatings were then submerged in an ethanolic solution of myristic acid, MA, to produce a superhydrophobic, SHP, surface. The Raman spectra demonstrated that the generated graphene was of high quality. Fourier transform infrared spectroscopy findings confirm the modification of the Ni@G coating by MA, Ni@G@MA, and the modification of the Ni@Cr-G composite with MA, Ni@Cr-G@MA. The results of the scanning electron microscope revealed that the created SHP coatings have nanoscale features. The wettability results showed that the water contact angle values for Ni@G@MA and Ni@Cr-G@MA coatings are 158° and 168°, while the water sliding angle values for both coatings are 4.0 ^o^ and 1.0°, respectively. The atomic force microscopy results show that both Ni@G and Ni@Cr-G coatings increase the roughness of the steel. The chemical and mechanical stability of the Ni@Cr-G@MA coating was higher than those of the Ni@G@MA coating. The coated steel by Ni@Cr-G@MA exhibits UV stability up to 110 h, while the SHP-coated steel by Ni@G@MA exhibits UV stability for 60 h. The potentiodynamic polarization results show that the value of the corrosion current density for bare steel is 13 times that of steel coated with Ni@G@MA, and 21 times that of coated steel with Ni@Cr-G@MA. The electrochemical impedance spectroscopy, EIS, results show that the charge transfer resistance for steel coated with Ni@G@MA is 38 times that of bare steel, while steel coated with Ni@Cr-G@MA is 57 times that of bare steel. Potentiodynamic polarization and EIS results show that the SHP Ni@Cr-G@MA film exhibits higher corrosion resistance than Ni@G@MA film.

## Introduction

The wettability of solid surfaces is a topic of growing interest from both a theoretical and practical perspective^1^. Artificial superhydrophobic, SHP, materials with sufficient surface roughness and low surface energy have successfully been produced inspired by the lotus effect^1,2^. SHP surfaces with water contact angles more than 150° and water slide angles less than 10° have acquired extensive attention due to their vast array of potential uses, such as anticorrosion^3^, oil–water separation^4^, self-cleaning^5^, anti-icing^6^, antifouling^7^, biomedicine^8^, and drag reduction^9^. Various methods were utilized to manufacture bio-inspired SHP surfaces, including chemical vapor deposition^10^, chemical etching^11^, 3D printing^12^, sol-gel^13^, electrospinning^14^, anodization^15^, spraying^16^, and electrodeposition^17^. Most of these procedures are intricate and time-consuming, restricting their industrial applications on a large scale. In contrast, electrodeposition technology has been extensively utilized in the preparation of SHP surfaces due to its advantages, including scalability, low cost, ease of control, simplicity, fabrication of a robust SHP coating, and by altering factors like the deposition time, current, and voltage, the surface morphology of the coating may be readily manipulated^18–23^.

Carbon steel is the construction material most frequently used in many industries as its great mechanical qualities and low price. It is used extensively in machinery for processing metals, construction, bridges, chemical processing, petroleum production, and marine applications^24–26^. Under these circumstances, steel corrosion and its suppression are challenging process issues. Corrosion is one of our society's most serious issues because of its impact on both the economy and public safety^27–29^. The creation of SHP coatings, which significantly boost steel corrosion resistance, is one of the most crucial strategies to minimize the corrosion of steel^30,31^.

Nickel is a primary industrial metal with desired characteristics, for instance, magnetism, hardness, and corrosion resistance. When nickel is applied to steel, the nickel deposited reduces bare steel's corrosion. When paired with superhydrophobicity, the deposited nickel coating can provide special benefits, including self-cleaning and better corrosion resistance^32^. Graphene is categorized as a nanomaterial with a two-dimensional structure composed of carbon atoms resembling a honeycomb lattice^33^. Graphene exhibits exceptional thermal and electrical conductivity, impermeability, significant nonlinear diamagnetism, and great optical transparency^34,35^. In addition to its unique features, graphene is the most promising material for applications such as electrochemical energy storage, water purification, supercapacitors, biomedical implants, gas detection, and thin protective coatings^36,37^. Many techniques are used for manufacturing graphene, including single-crystal cleavage, electrochemical or chemical exfoliation, chemical vapour deposition, and annealing^38–40^. In contrast, most environmentally friendly synthesis processes utilize fewer hazardous chemicals and natural resources. We have developed a green manufacturing technique for synthesizing graphene from banana leaves for the first time.

The leaves of bananas are big, wide, and elongated and they are among the most valuable agricultural crops in terms of metric tons harvested. Banana leaves are typically burned or disposed of in a way that could pollute the environment. Due to the high levels of carbon (43.5%) present, this waste can be used successfully in the production of graphene which is then utilized to form a SHP film on steel surfaces for corrosion inhibition^41–43^.

SHP surfaces have a limited range of practical uses due to their low mechanical durability and chemical instability^44,45^. SHP surfaces must improve their chemical stability and mechanical abrasion resistance to be used in industrial applications.

In this work, for the first time, a facile and environmentally friendly approach was used for the production of high-quality graphene from biomass waste of banana leaves. Two rough coats of Ni-graphene, Ni@G, and Ni-graphene doped with chromium, Ni@Cr-G, were fabricated on steel substrates by electrostatic deposition. These coatings were then immersed in an ethanolic solution of myristic acid, MA, to produce SHP surfaces. Innovatively, we use graphene synthesized from banana leaves and a composite of Cr-G as an additive to enhance the mechanical and chemical stability of the SHP coatings. Improving the mechanical durability and chemical stability of SHP coatings is a crucial factor to use them in different industrial applications. We use myristic acid as a low surface energy compound because it is inexpensive compared to other low surface energy materials such as silanes and toxic fluorinated polymers as well as it is an environmentally friendly substance. The wettability, chemical and mechanical stability, UV resistance, and corrosion performance were evaluated for the manufactured SHP coatings in 0.5 M NaCl solution.

## Experimental

### Materials

As a substrate, a steel plate with the dimensions 2.0 cm, 1.0 cm, and 0.1 cm was used in this study. The banana leaves were collected in accordance with institutional, national, and international guidelines and legislation. Sodium chloride, sulfuric acid, boric acid, nickel sulfate, nickel chloride hexahydrate, anhydrous ethanol, potassium hydroxide, chromium sulfate pentahydrate, myristic acid, and sodium hydroxide of analytical quality were utilized.

### Graphene production from banana leaves

The banana leaves are thoroughly cleaned and then burned for about an hour at 250 °C to produce banana leaves ash, BLA. BLA and potassium hydroxide are combined in a crucible with a wt% ratio of 1:5 for BLA: KOH. The sample was annealed at 700 °C for 3.0 h in a muffle furnace. The sample was repeatedly rinsed in distilled water to eliminate extra potassium hydroxide before being dried overnight hours at 100 °C.

CrSO4.5H_2_O was dissolved in 100 mL of deionized water comprising BLA with a wt% ratio of 1:20 for Cr: BLA. The mixture was sonicated for 30 min, followed by 1 h of stirring. The mixture was then dried in an oven overnight at 60 °C. The mixture was activated by mixing the mixture with KOH in a crucible with a wt% ratio of 1:5 for mixture: KOH. The chromium-modified graphene, Cr-G, was produced by pyrolyzing the mixture for three hours at 700 °C in a muffle furnace. The sample was then thoroughly rinsed in distilled water to eliminate any remaining KOH, followed by a 24-h drying period at 100 °C.

### Superhydrophobic coating manufacture

Prior to electrodeposition, the steel was polished using abrasive paper of varying grades, starting with rough (grade 300) and evolving to smooth (grade 1000). The steel was immersed in a solution of soap for 10 min, then rinsed with distilled water, submerged in 2.0 M H_2_SO_4_ for one minute, and then immediately placed into the bath for electrodeposition. The criteria for electrodeposition for coating the steel substrate with nickel modified by graphene, Ni@G, and the nickel modified by Cr-G, Ni@Cr-G, are shown in Table 1. The steel substrate, which served as the cathode, was separated from the platinum sheet, which served as the anode, by a distance of 2.0 cm. Both Ni@G and Ni@Cr-G coatings were cleaned with distilled water and allowed to air dry for 24 h. The steel substrates coated by Ni@G and Ni@Cr-G were submerged for 0.25 h in ethanolic solutions containing 0.01 M myristic acid, MA, then washed with ethanol and permitted to dry overnight at room conditions. The as-prepared Ni@G coating modified with MA, Ni@G@MA, and the Ni@Cr-G coating modified with myristic acid, Ni@Cr-G@MA, were exposed to several characterizations and assessment processes.

**Table 1:** Bath components and working environments for electrodeposition of Ni@G@MA and Ni@Cr-G@MA coating on the steel surface.

Factor	Level
Source of nickel ions	
NiCl_2_·6H_2_O	40 g L^−1^
NiSO_4_	176 g L^−1^
pH buffer	
H_3_BO_3_	60 g L^−1^
SLS	0.4 g L^−1^
G or Cr-G	0.4 g L^−1^
Time of deposition	6.0 min
Deposition potential	11.0 V

### Surface characterization

Raman spectra of graphene and modified graphene with chromium were gained utilizing a spectrometer (Senttera-Broker) supplied with a 532 nm wavelength laser. A Fourier transform infrared spectrophotometer, FTIR, was used to analyze the surface's chemical composition (model: Bruker Tensor 37 FTIR). An X-ray diffractometer was applied to perform an X-ray diffraction examination utilizing monochromatic Cu K radiation (Bruker D2 phaser).

The scanning electron microscope, SEM, was employed to investigate the surface morphology of the produced SHP coatings (model JSM-200 IT, JEOL). The atomic force microscopy, AFM, was accomplished by Scanning Probe Microscope (SPM9600-Shimadzu Japan). The WCA and WSA were computed by means of an optical contact angle goniometer and 5 µL water droplets (Rame-hart CA instrument, model 190-F2). The presented WCA and WSA values are the averages of three measurements taken at varied substrate locations.

### Chemical stability

The generated SHP films were submerged in solutions with varying pH (pH = 1–13) for 1 h, and the WCA and WSA were evaluated at each pH^46,47^. Sulfuric acid and sodium hydroxide were employed to change the pH of the solution. The presented results of chemical stability are the averages of three tests at varied substrates.

### Mechanical abrasion

The scratch and sand impact experiments have been applied to test the mechanical properties of the manufactured SHP films. The scratch test was accomplished by applying the SHP film to a SiC paper as an abrasion surface (800 grade). A 5.0 kPa pressure was applied to the SHP film. For each 100 mm of abrasion, the WCA and WSA of a water droplet on the prepared SHP films were measured. In the sand impact test, SHP-coated steel was struck by 50 g of sand that had been dropped from a height of 50 cm. The WCAs and WSAs for every 50 g of sand that hit the SHP surface were measured to assess the sample's water Superhydrophobicity. The presented mechanical stability results are the averages of three measurements at varied substrates.

### UV resistance tests

The produced SHP surface was tested for UV resistance by measuring its wettability during different time intervals of UV irradiation (λ = 365 nm, 300 W). The WCA and WSA values were calculated every two hours. The coating and UV lamp are still around 10 cm apart. The presented UV resistance results are the averages of three measurements at varied substrates.

### Corrosion test

The electrochemical experiments were carried out using ACM frequency response analyzer and a three-electrode cell. A graphite rod worked as the counter electrode, while an Ag/AgCl electrode acted as the reference electrode. The bare and coated steel with Ni@G@MA and Ni@Cr-G@MA films were employed as the working electrodes. The working electrodes were covered with an epoxy coating, leaving a 1 cm^2^ area exposed to the test solution. Before conducting electrochemical experiments, the working electrode was put into a 0.5 M NaCl solution-filled cell and left there for 30 min at room temperature to attain the rest potential. The frequency range and signal amplitude of electrochemical impedance spectroscopy, EIS, measurements were 0.01 ≤ f ≤ 1.0 × 10^4^ and 10 mV around the open circuit potential, respectively. Potentiodynamic polarization, PDP, measurments were done at a scan rate of 30 mV/min with a potential range of ± 250 mV around the open circuit potential. The experiments were twice-examined to confirm that the measurements were precise and the error was within 2%.

## Results and discussion

### Raman spectra

**Figure 1:**
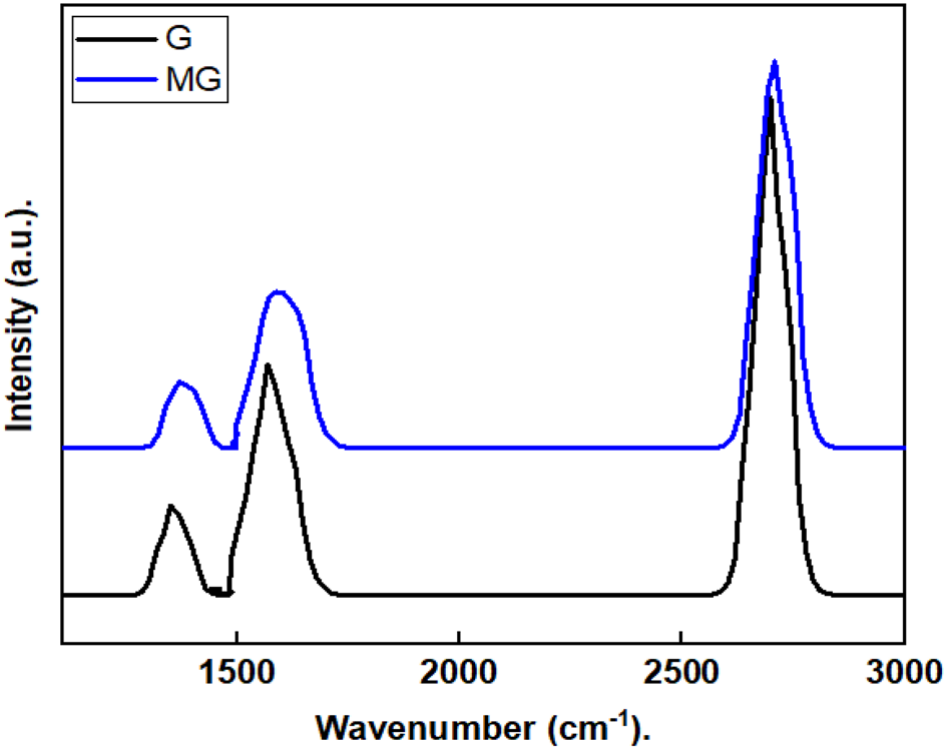
Raman spectra of the graphene, G, and modified graphene, MG.

Raman scattering is undoubtedly a useful tool to deliver a non-destructive technique to analyze the ordered and disordered crystallographic structure ^48^. Even on the strongly interacting transition metal surface, Raman spectroscopy is a valuable tool for characterizing graphene^49^. The graphene and modified graphene Raman spectrum are shown in Fig. 1. The D peak for graphene is produced at 1350 cm^−1^ due to the sp2 atom's breathing mode, this is active when graphene contains defects and impurities ^50^. However, the E2g phonon of carbon atoms that have undergone sp2 hybridization is what causes the G peak at 1571 cm^−1^. Furthermore, graphene displays a significant 2D peak at about 2692 cm^−1^. The shape, location, and peak strength concerning the D band are strongly influenced by the number of graphene layers. A sharp and high-intensity 2D peak demonstrated that graphene was effectively produced^51^. The Raman spectrum of the modified graphene shows a blue shift in frequency for G (1370 cm^−1^), D (1589 cm^−1^), and 2D (2707 cm^−1^) peaks due to the contact of graphene with chromium. This result coincides with the previous report on graphene-chromium composite^49^.

### FTIR results

**Figure 2:**
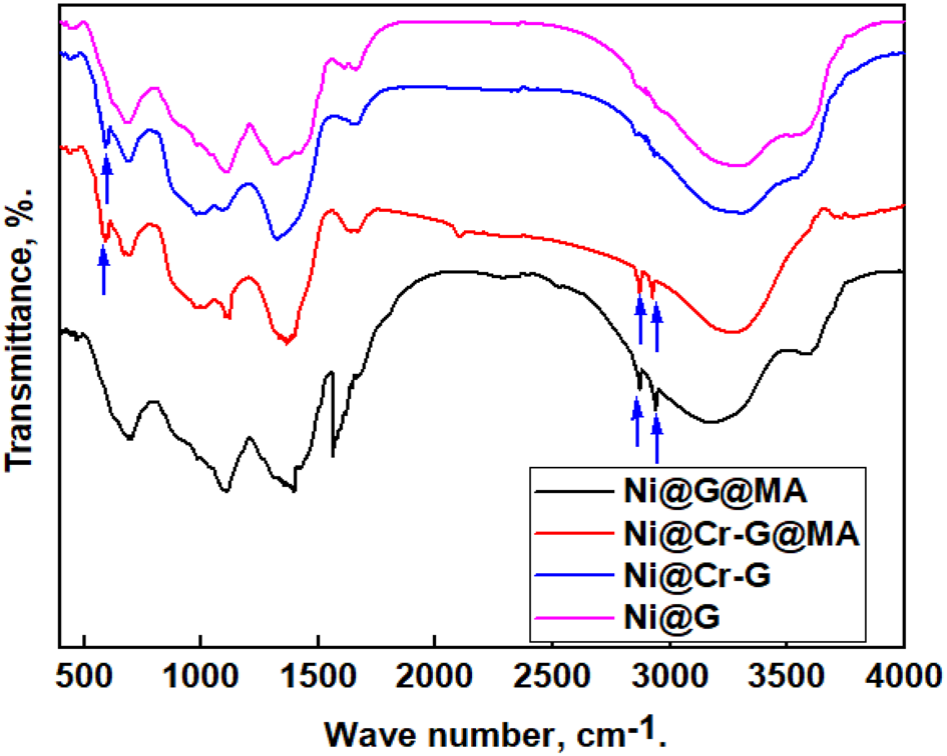
FTIR spectra of the coated steel with Ni@G, Ni@Cr-G, Ni@G@MA, and Ni@Cr-G@MA.

The FTIR spectra of coated steel by Ni@G, Ni@Cr-G, Ni@G@MA, and Ni@Cr-G@MA are displayed in Fig. 2. The spectra of steel treated with Ni@G illustrates the graphene's distinctive peaks. The peak at 3278 cm^−1^ is due to the O–H bond vibration, complemented by the C–OH band at 1105 cm^−1^ due to the hydroxyl groups of the graphene^3^. The peak at 1660 cm^−1^ is attributed to the stretching vibration of C=C and C=O groups^52^. The peak at 1342 cm^−1^ is due to the O–H bending or C–O stretching vibration^53^. The peak at 677 cm^−1^ is due to Ni(OH)_2_^30^. The spectra of steel treated with Ni@Cr-G display the same peaks of Ni@G and an additional peak at 590 cm^−1^, which is attributed to Cr confirming the doping of graphene with chromium^54^. The peaks at 2922 cm^−1^ and 2852 cm^−1^ are responsible for the asymmetry and symmetry vibration of the myristic acid CH_2_– in the spectrum for coated steel with Ni@G@MA and Ni@Cr-G@MA suggesting that the deposited Ni@G, Ni@Cr-G are modified by myristic acid, respectively^17^.

### XRD results

**Figure 3:**
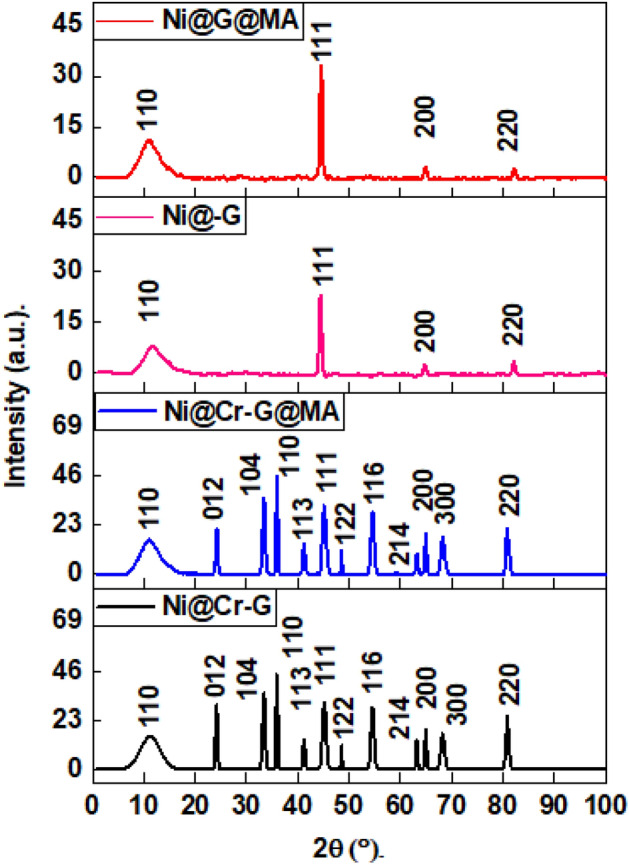
XRD patterns of SHP coated steel by Ni@G, Ni@Cr-G, Ni@G@MA, and Ni@Cr-G@MA.

The crystal orientation and composition of coated steel with Ni@G, Ni@Cr-G, Ni@G@MA, and Ni@Cr-G@MA films were revealed using the XRD technique, Fig. 3. The Ni@G coat exhibits four diffraction peaks in its XRD pattern. The three peaks at 2Ɵ values equal 43.9°, 65.1°, and 81.9° are associated with faced cubic centered, fcc, of NiO (JCPDS # 47–1049)^3^. The XRD peak at 2Ɵ values equals 11.1°, corresponding to graphene^55,56^. The XRD pattern of the Ni@G@MA coat is similar to that of the Ni@G coat, showing that the grafting of myristic acid on the Ni@G coat has no impact on the crystal orientation. The broad graphene peak demonstrates the material's small particle size. The XRD patterns of Ni@Cr-G exhibit identical diffraction peaks of Ni@G that are related to the deposition of fcc NiO and graphene with an additional 8 diffraction peaks. The additional 8 diffraction peaks are located at 2Ɵ values equal to 24.1°, 33.5°, 35.8°, 41.4°, 48.5°, 54.4°, 63.1°, and 68.6° are associated with Eskolaite chromium (III) oxide phase (Cr_2_O_3_) (JCPDS # 038‐1479)^57^. The XRD patterns of Ni@Cr-G@MA show comparable diffraction peaks to Ni@Cr-G, demonstrating that the grafting of myristic acid on the Ni@Cr-G coat has no impact on the crystal orientation.

### SEM and wettability results

**Figure 4:**
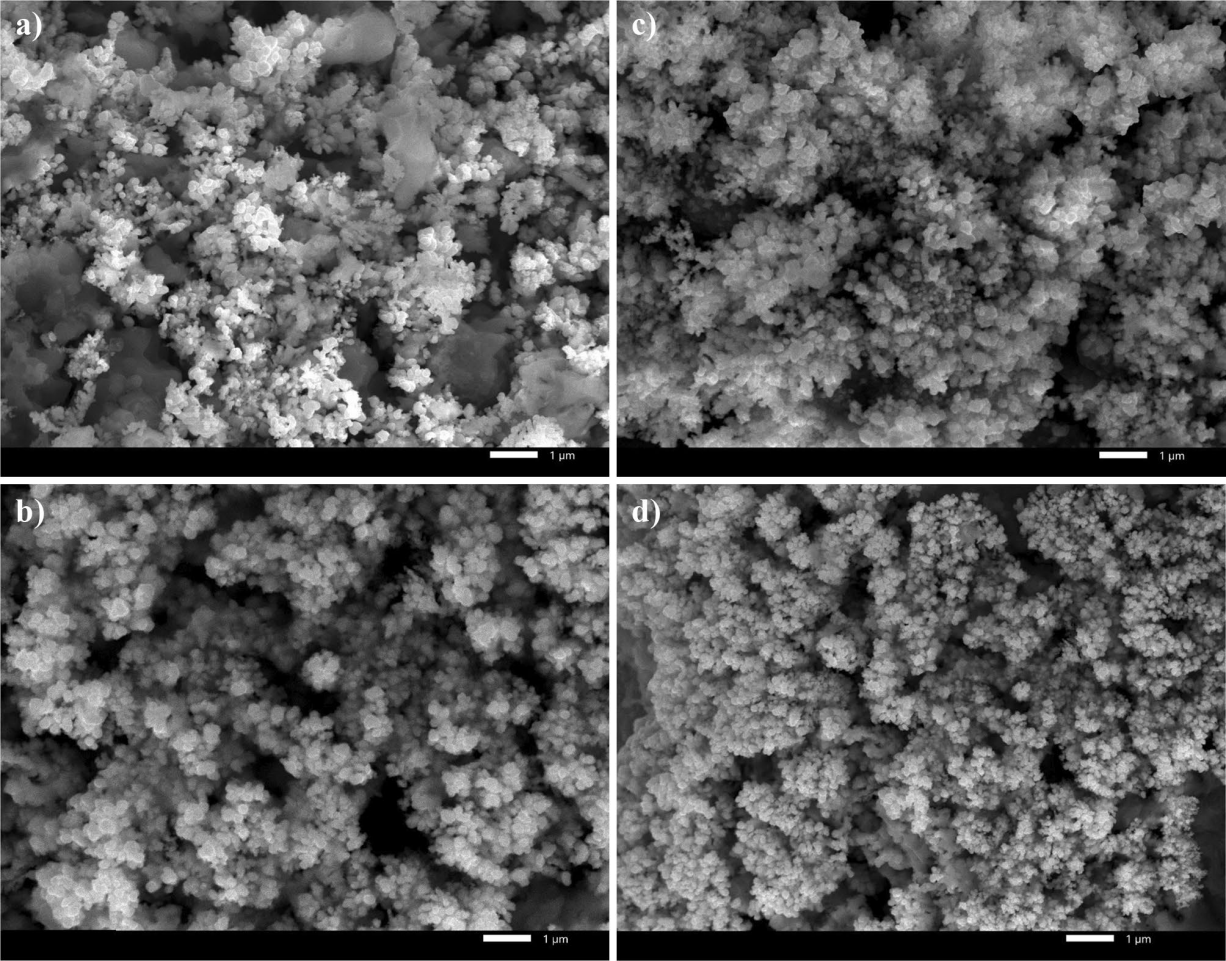
SEM micrographs of coated steel by (**a**) Ni@G, (**b**) Ni@G@MA, (**c**) Ni@Cr-G, and (**d**) Ni@Cr-G@MA.

Surface morphology is an important factor to consider when examining SHP characteristics; hence the morphology of the generated SHP films on the steel surface has been examined using the SEM technique. The micrograph of steel coated by Ni@G is shown in Fig. 4a, and it is obvious that the electrodeposited layer features micro-nano circular shapes. The micrograph of steel coated with Ni@G@MA is shown in Fig. 4b. It demonstrates that the morphology of the deposited structures remains unaltered by grafting the Ni@G coat with myristic acid.

Figure 4c displays the micrograph of coated steel with Ni@Cr-G. It is obvious that the electrodeposited layer has micro-nano circular shapes, and the density of the electrodeposited structures is higher than that of Ni@G; consequently, it will have higher surface roughness. Figure 4d displays the micrograph of steel coated with Ni@Cr-G@MA; the grafting of the covering Ni@Cr-G layer with myristic acid does not change the morphology of the electrodeposited structures.

**Figure 5:**
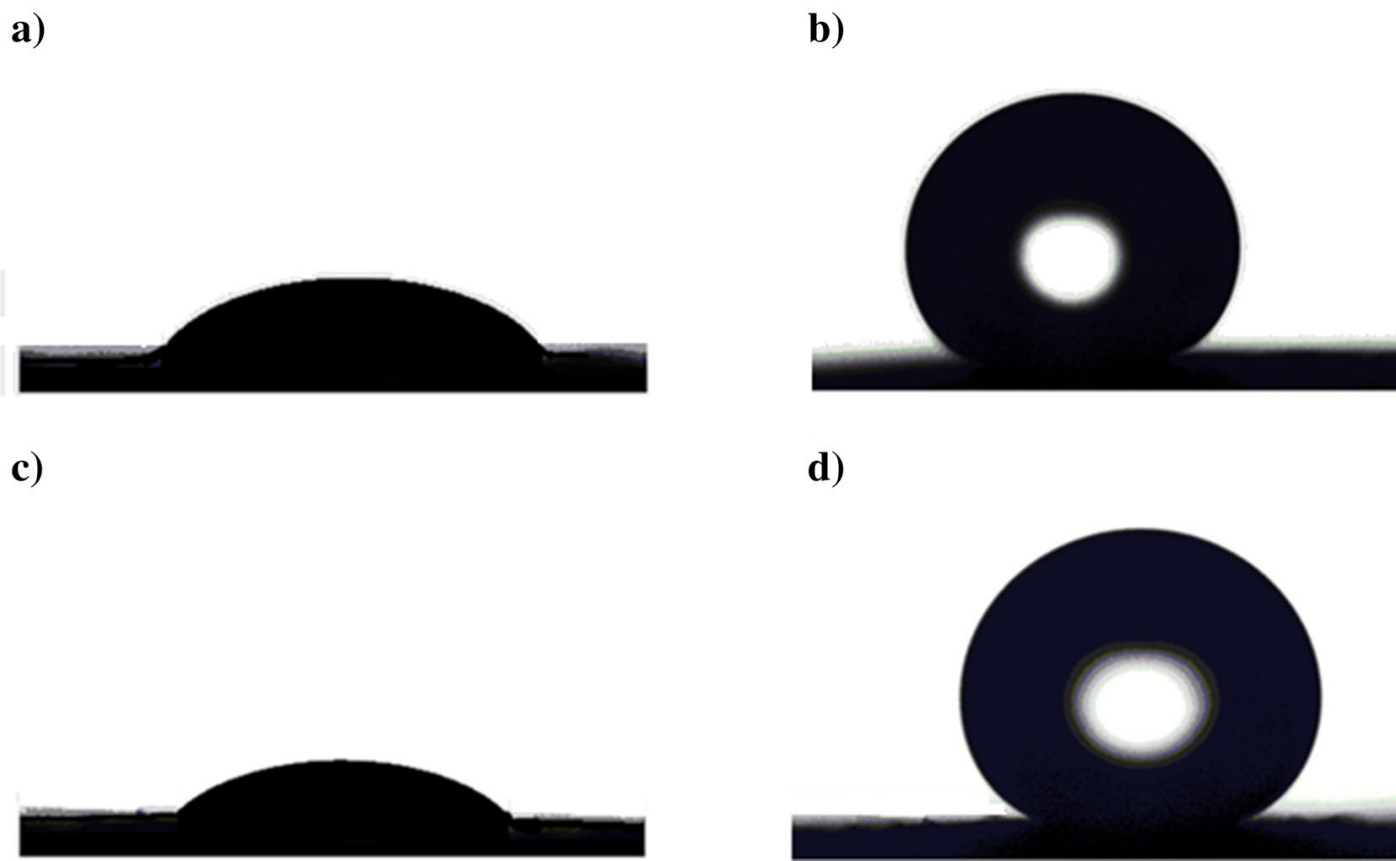
The shape of a water droplet on coated steel by (**a**) Ni@G, (**b**) Ni@G@MA, (**c**) Ni@Cr-G, and (**d**) Ni@Cr-G@MA.

The coated steel by Ni@G and Ni@Cr-G have WCAs of 44° and 31°, respectively, but the water droplet does not slide on them. Since increasing surface roughness improves the hydrophilicity of hydrophilic surfaces, and improves the hydrophobicity of hydrophobic surfaces. So, doping of hydrophilic Ni@G coat with Cr will enhance the surface roughness and so the hydrophilic characteristics will be improved and the contact angle decreases^58^. The WCA and WSA values for Ni@G@MA film are 158° and 4°, respectively, while they are 168° and 1° for Ni@Cr-G@MA. The shape of a water droplet on the bare and coated steel is depicted in Fig. 5. These results imply that the doping of graphene with chromium at the steel surfaces enhances the surface roughness, so the SHP characteristics are improved^19^.

### AFM results

**Figure 6:**
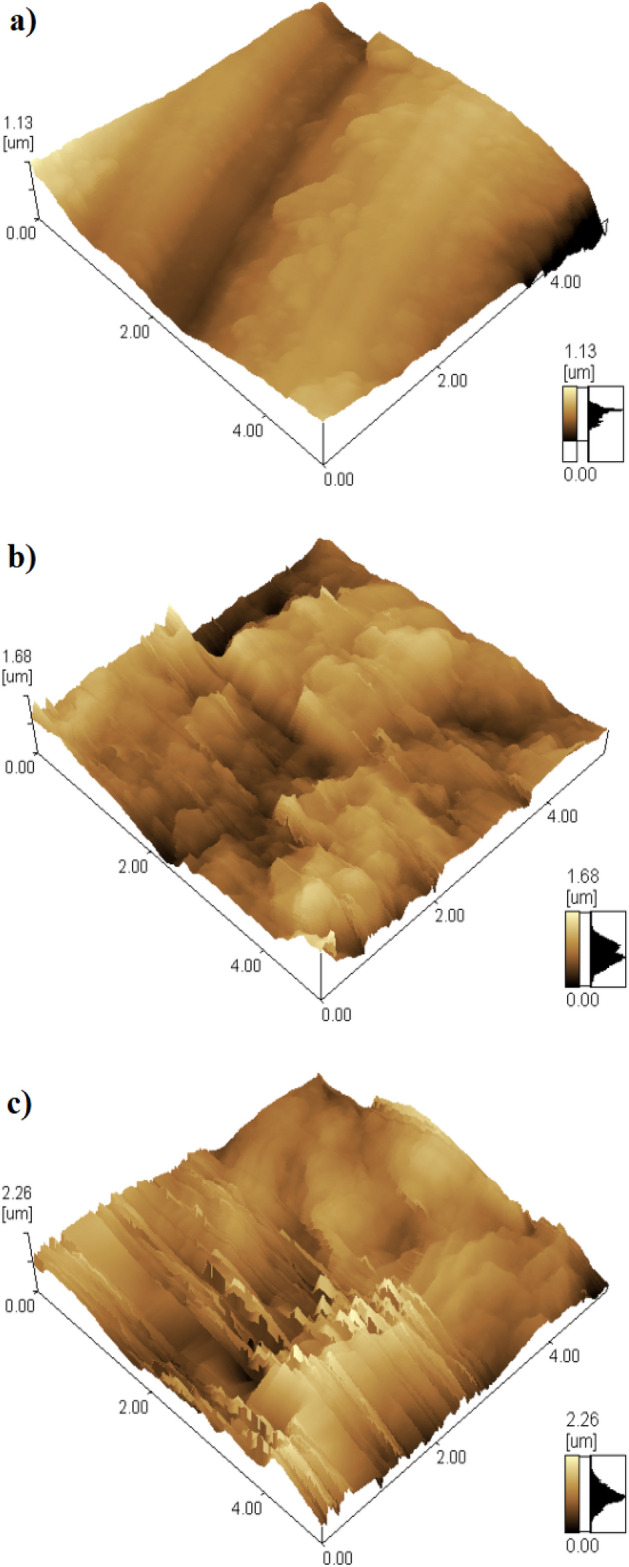
3D AFM topography images of the surface of (**a**) bare and SHP coated steel with (**b**) Ni@G@MA and (**c**) Ni@Cr-G@MA.

The surface roughness of the bare and SHP coated steel was further characterized using the AFM. The arithmetic average roughness, Ra, of the bare steel was 0.79 µm according to the 3D AFM image, Fig. 6a. The Ra value increased to 1.74 µm for SHP coated steel by Ni@G@MA, indicating that the deposited coat increases the steel surface roughness, Fig. 6b. While for SHP coated steel by Ni@Cr-G@MA, the Ra value increased to 2.41 µm, Fig. 6c, which may be ascribed to the doping of the graphene with chromium which greatly increases the roughness of the steel surface.

### Chemical stability

**Figure 7:**
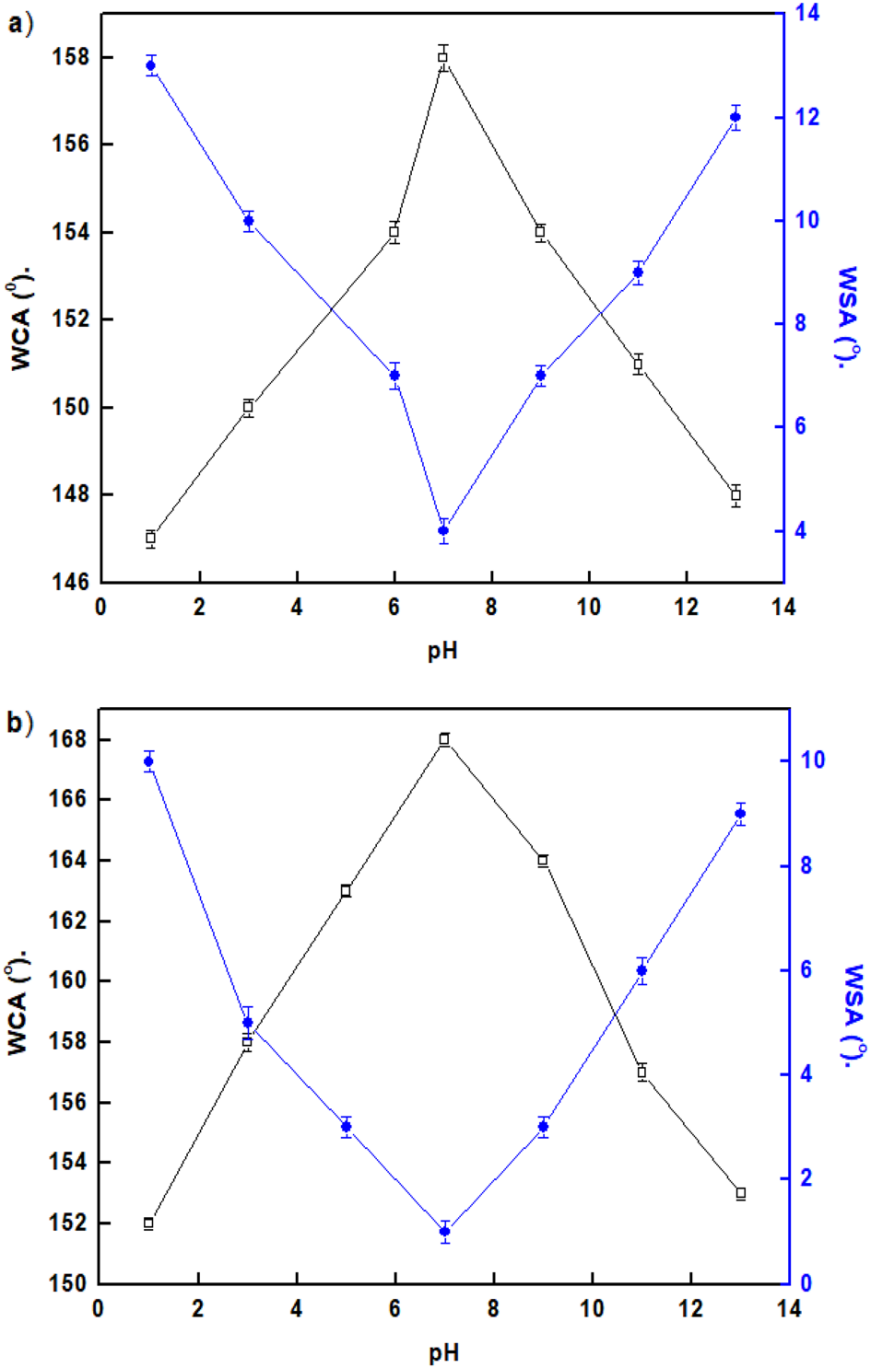
Variation of solution pH and the WCAs and WSAs of the coated steel with (**a**) Ni@G@MA and (**b**) Ni@Cr-G@MA.

Chemical stability is a crucial prerequisite for the long-lasting performance of SHP coatings in harsh solution conditions. Figure 7 shows the relationships between the solution pH and the WCAs and WSAs of water droplets on the SHP coatings.

**Figure 8:**
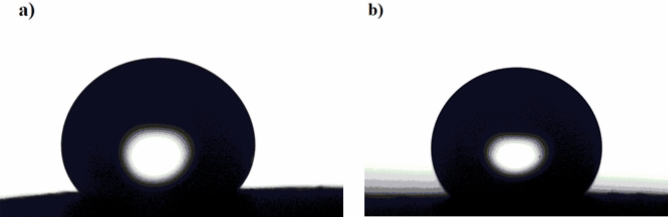
The shape of a water droplet on SHP coated steel by (**a**) Ni@G@MA, and (**b**) Ni@Cr-G@MA after submersion in a solution of pH 1.0 for one hour.

The results show that in the pH range of 3–11, Ni@G@MA films are SHP, whereas Ni@Cr-G@MA films are SHP in the pH range of 1–13, where the WCAs are often greater than 150°, and the WSAs are lower than 10°. The SHP coating's chemical stability in both basic and acidic environments is therefore improved by adding Cr to G. The shape of the water droplet on the SHP coated steel after immersion for one hour in a solution of pH 1.0 is shown in Fig. 8. The SHP coated steel with Ni@Cr-G@MA has comparable chemical stability to that of the previously reported value^59^, and it has greater chemical stability than several previously known values^60–63^.

### Mechanical stability

**Figure 9:**
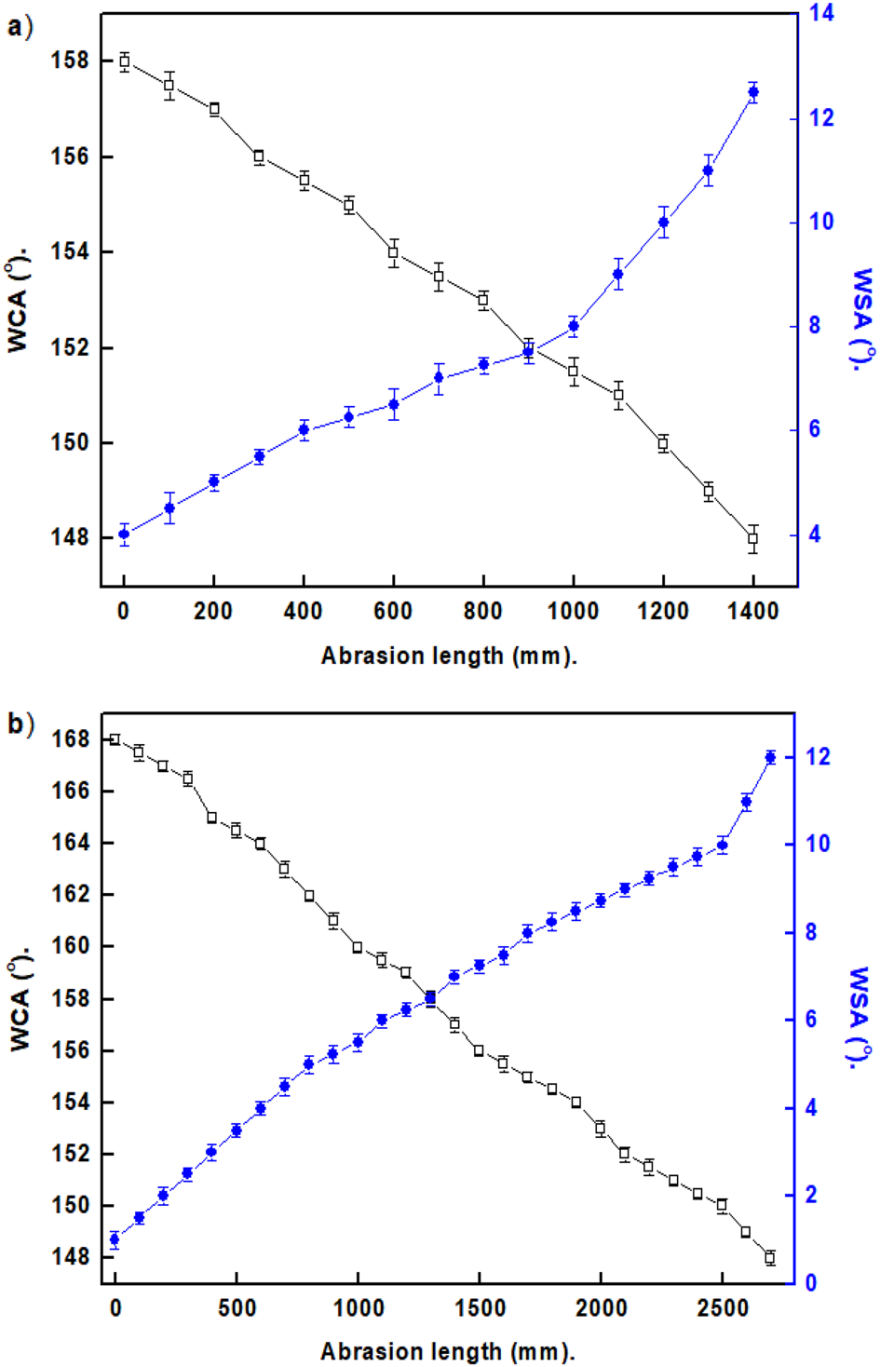
The change in WCAs and WSAs against the abrasion length for coated steel with (**a**) Ni@G@MA and (**b**) Ni@Cr-G@MA.

Because they are typically mechanically fragile, SHP surfaces have limited practical uses. Some surfaces with SHP properties can crack when touched with a finger^3^. The scratch and sand impact tests were used to assess the created SHP films' resistance to mechanical abrasion. The changes in WCAs and WSAs of the produced SHP films with the abrasion length are shown in Fig. 9. The prepared Ni@G@MA SHP film holds its superhydrophobicity until a 1200 mm abrasion length.

**Figure 10:**
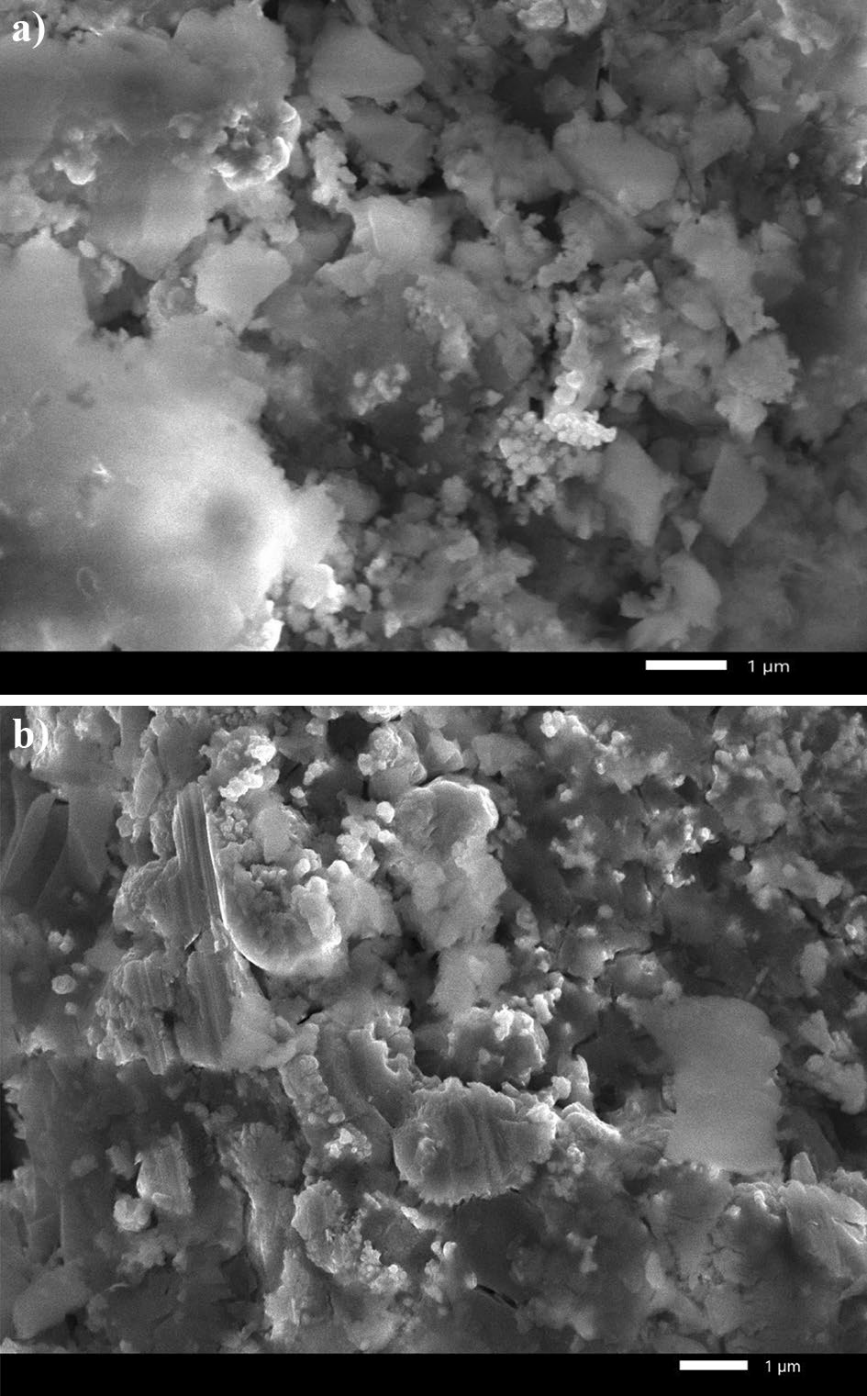
The SEM micrographs of SHP coated steel by (**a**) Ni@G@MA and (**b**) Ni@Cr-G@MA after abrasion test.

**Figure 11:**
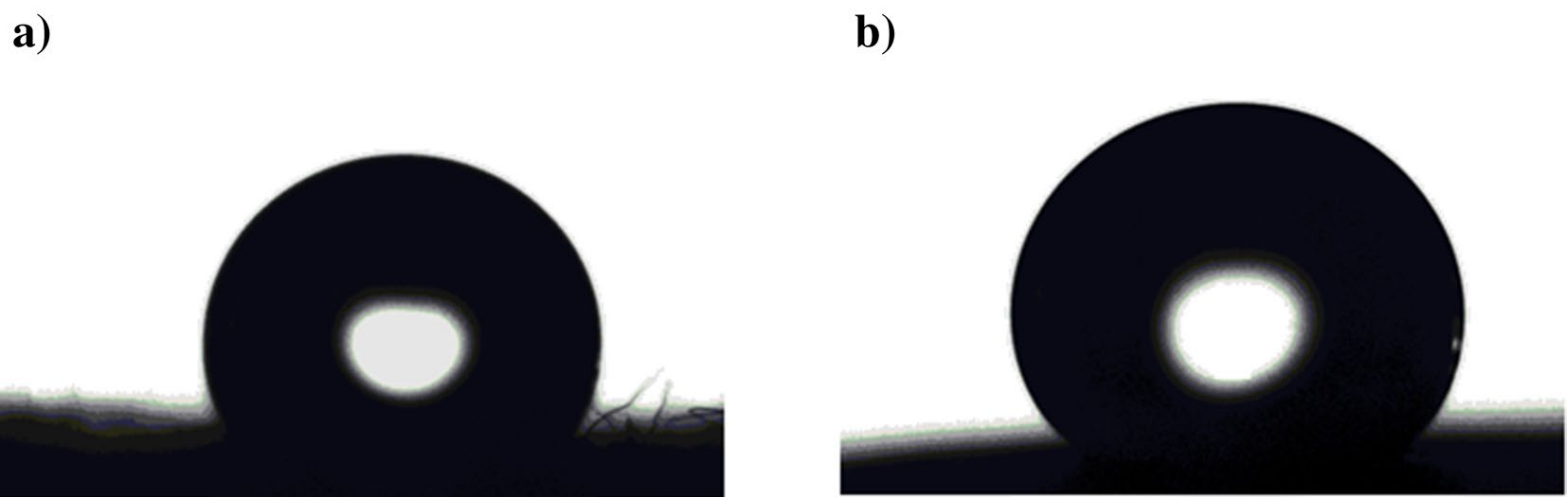
The shape of a water droplet on SHP coated steel by (**a**) Ni@G@MA, and (**b**) Ni@Cr-G@MA after the mechanical abrasion test.

In comparison, the prepared Ni@Cr-G@MA SHP film sustains its superhydrophobicity until a 2500 mm abrasion length. These findings demonstrated that adding Cr significantly improved the produced SHP G-based film's mechanical stability. Figure 10 shows the SEM micrographs of coated steel with Ni@G@MA and Ni@Cr-G@MA after the abrasion test. The Figure demonstrates that the micro-nano circular shape structures of the prepared SHP coatings were destroyed. Since the surface roughness and low surface energy are the two crucial prerequisites for the fabrication of the superhydrophobic coating, so the destroying of the micro-nano circular particles will greatly reduce the surface roughness, and consequently, the manufactured coatings lose their superhydrophobic properties. The shape of the water droplet on the SHP coated steel after the abrasion test is shown in Fig. 11. The coated steel with Ni@Cr-G@MA has higher abrasion resistance than several previously reported values ^63–68^. However, the coated steel with Ni@Cr-G@MA has a lower abrasion resistance than a few previously reported value^69,70^.

**Figure 12:**
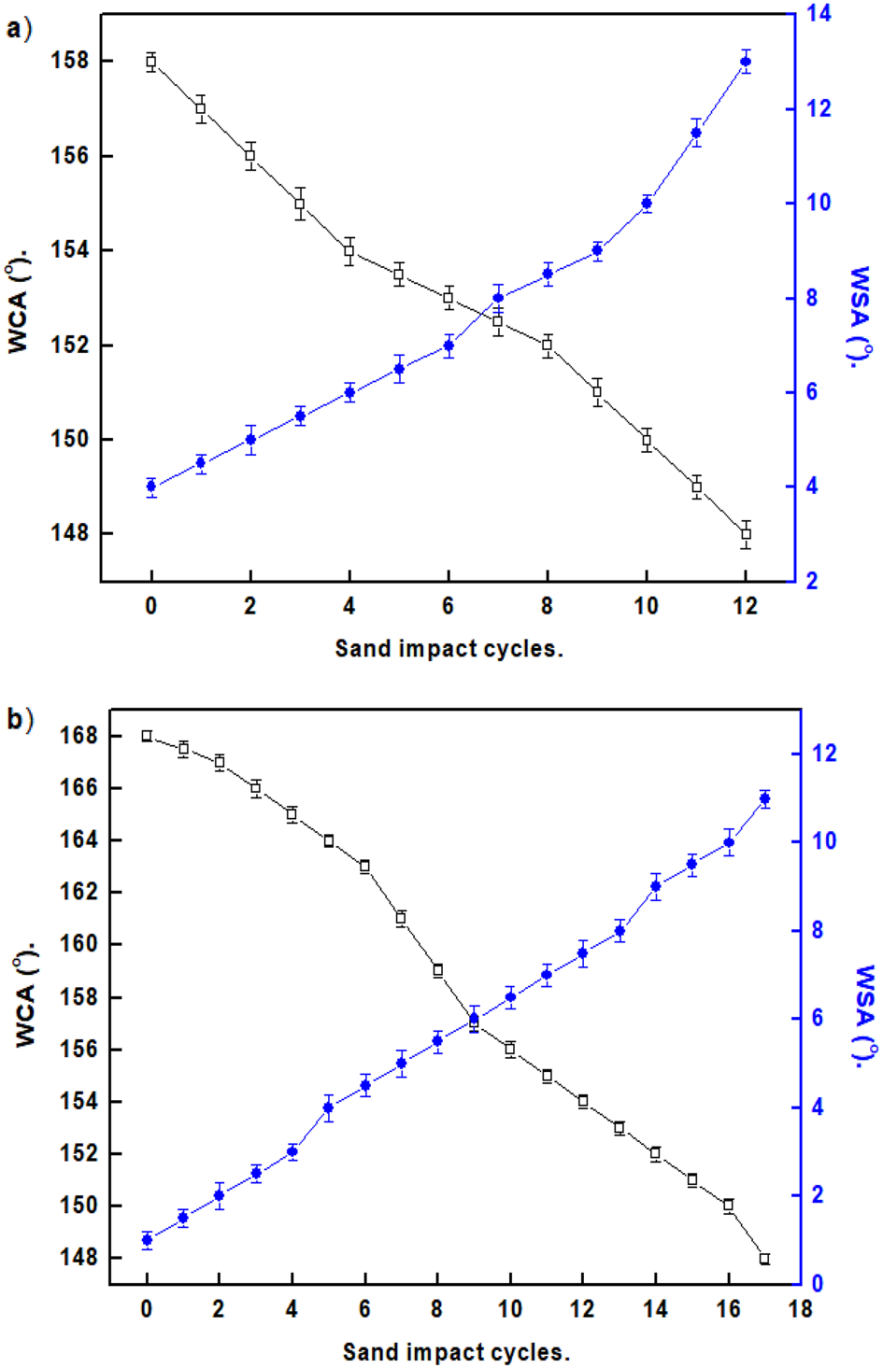
The effect of sand impact cycles on the WCAs and WSAs of coated steel with (**a**) Ni@G@MA and (**b**) Ni@Cr-G@MA.

The sand abrasion tests were performed to assess the mechanical performance of the SHP coatings, as shown in Fig. 12. The Ni@G@MA film exhibits superhydrophobicity until 10 cycles of the sand impact, while the Ni@Cr-G@MA film maintains its Superhydrophobicity up to 16 cyclic of the sand impact. The Ni@Cr-G@MA has a sand impact resistance that is higher than many published values^62,71^.

### UV stability

**Figure 13:**
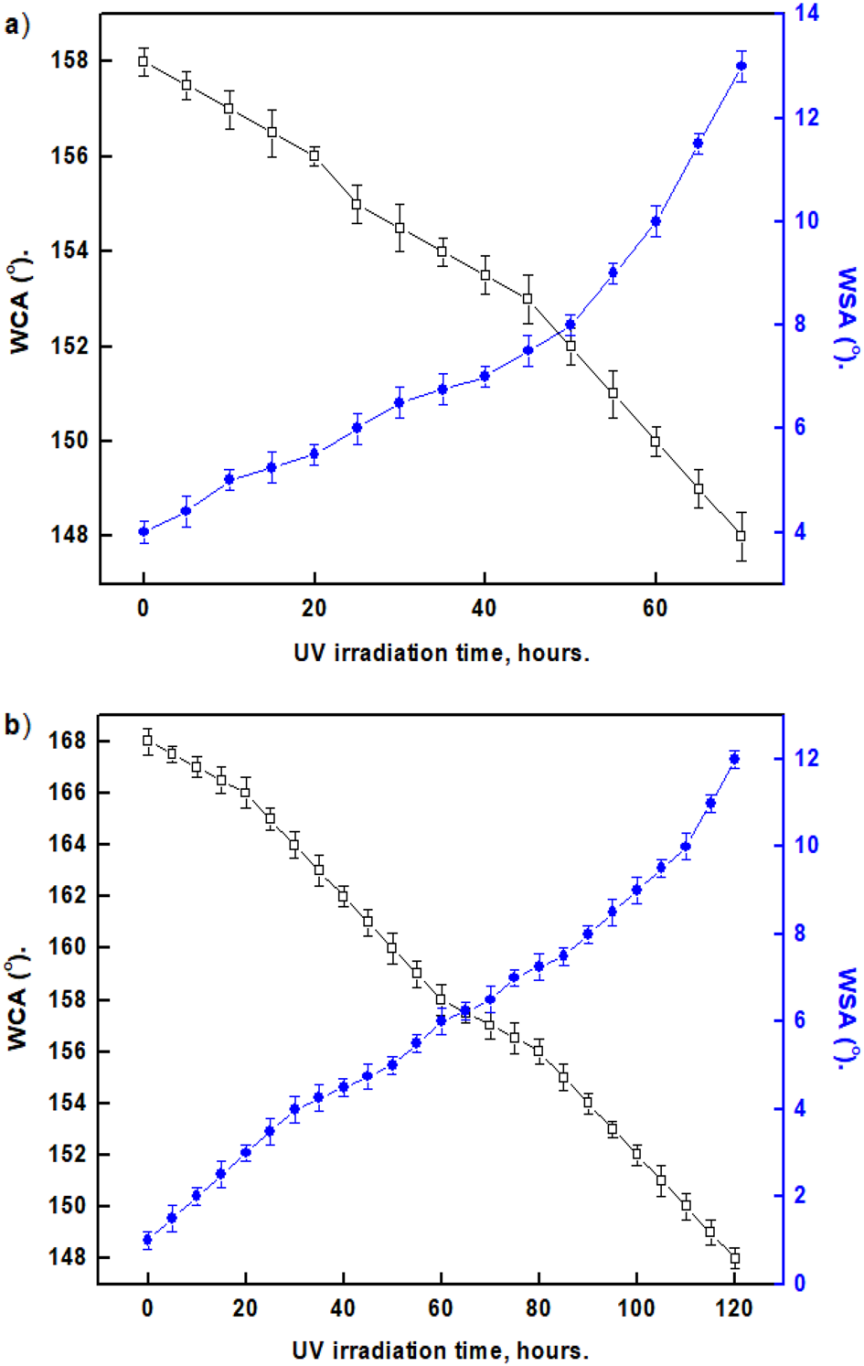
Effect UV-irradiation period on WCA and WSA of the coated steel by (**a**) Ni@G@MA and (**b**) Ni@Cr-G@MA.

**Figure 14:**
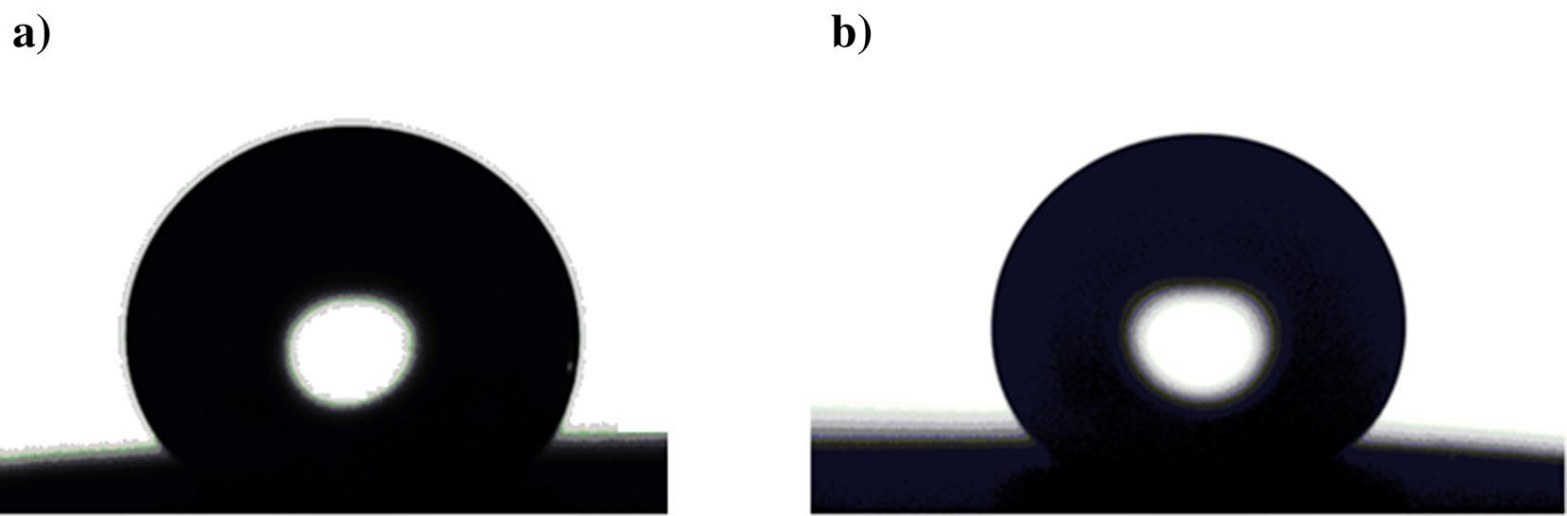
The shape of a water droplet on SHP coated steel by (**a**) Ni@G@MA, and (**b**) Ni@Cr-G@MA after the UV irradiation test.

An important consideration for outdoor applications is the production of coatings with UV resistance. When the right materials are chosen, a SHP surface can withstand prolonged UV exposure without losing its SHP properties. The effect of the UV-irradiation period on WCAs and WSAs of the SHP coated steel with Ni@G@MA and Ni@Cr-G@MA is shown in Fig. 13. While the coated steel with Ni@Cr-G@MA has UV stability for up to 110 h, the SHP coated steel with Ni@G@MA only has UV stability for 60 h. The shape of the water droplet on the SHP coated steel after exposure to the UV test is shown in Fig. 14. The SHP coated steel by Ni@Cr-G@MA has greater UV stability than several previously known values^72–76^.

### Corrosion resistance performance

#### PDP findings

**Figure 15:**
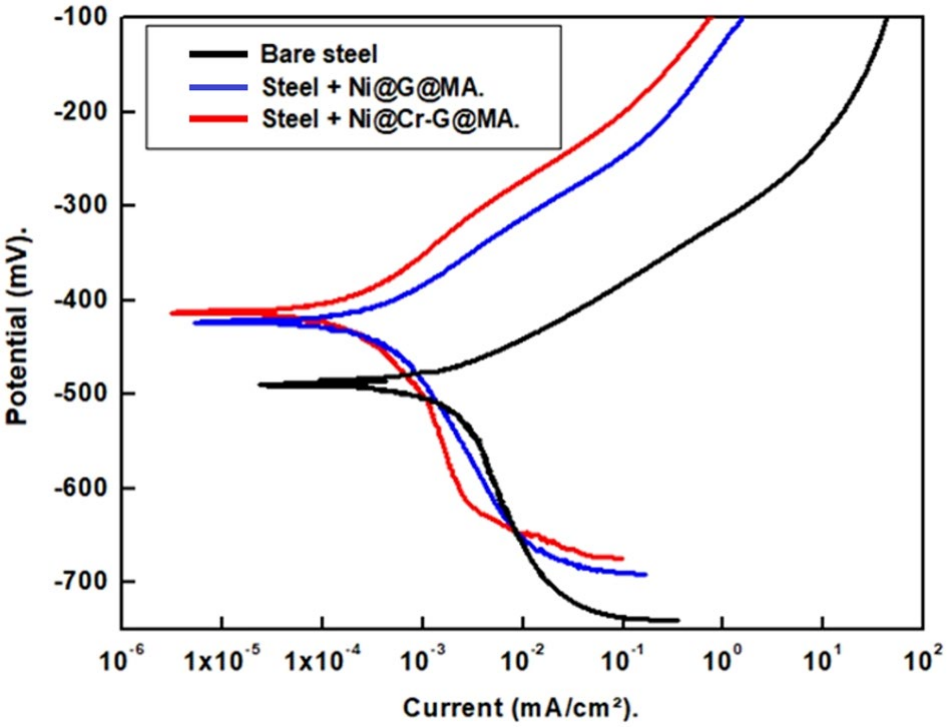
The PDP plots for the bare and the SHP coated steel in 0.5 M NaCl solution.

The corrosion behaviour of bare and SHP coated steel was studied by means of the PDP technique. The PDP plots of uncoated and SHP coated steel by Ni@G@MA and Ni@Cr-G@MA in an aqueous solution of 0.5 M NaCl are displayed in Fig. 15. The quick synthesis of corrosion products on the electrode surface for bare steel or the formation of a passive layer when the steel is treated with a SHP coating prevents the establishment of a perfect anodic Tafel region^77,78^. The cathodic polarization plots exhibit a limiting current, which suggests that the diffusion of the oxygen gas controls the cathodic process from the electrode surface to the bulk^79^. The limiting diffusion currents in the cathodic polarization curves represent the reduction reaction of oxygen, Eq. (1).

1 $${\text{O}}_{{2}} + {\text{2H}}_{{2}} {\text{O}} + {\text{4e}} \to {\text{4OH}}^{ - }$$

Table 2 shows the PDP parameters for bare and SHP coated steel, including corrosion potential (E_corr_), corrosion current density (i_corr_), and protection efficiency (% P). The protection efficiency was calculated using Eq. (2)^80^

2 $$\% {\text{P}} = \left[ {\left( {{\text{i}}_{{{\text{o}}. - }} {\text{i}}_{.} } \right)/{\text{i}}_{{{\text{o}}.}} } \right] \times {1}00$$

where, i_o._ and i are the corrosion current densities of the bare and SHP coated steel. The i_corr._ value for coated steel with Ni@G@MA is smaller than that for bare steel because of the coated steel's SHP characteristics. The steel coated with Ni@Cr-G@MA has the lowest i_corr._ value as its higher Superhydrophobicity. The SHP coating microstructures' trapped air can lower the contact area between the solution and steel, which causes a more rapid decrease in the i_corr_ value^81^. The contact area between the steel coated with Ni@Cr-G@MA and the solution is lower than that of the steel coated with Ni@G@MA, so the steel coated with Ni@Cr-G@MA has greater protection efficiency.

**Table 2:** The PDP parameters for the bare and the SHP coated steel in 0.5 M NaCl solution.

Deposit	− E_corr_ mV	β_a_ mV/decade	− β_c_ mV/decade	i_corr_ µA/cm^2^	%P
Bare steel	499.3	166.4	125.3	14.21	–
Steel + Ni@G@MA	426. 2	119.5	121.2	1.09	92.3
Steel + Ni@Cr-G@MA	409.1	102.2	119.4	0. 67	95.3

#### EIS findings

**Figure 16:**
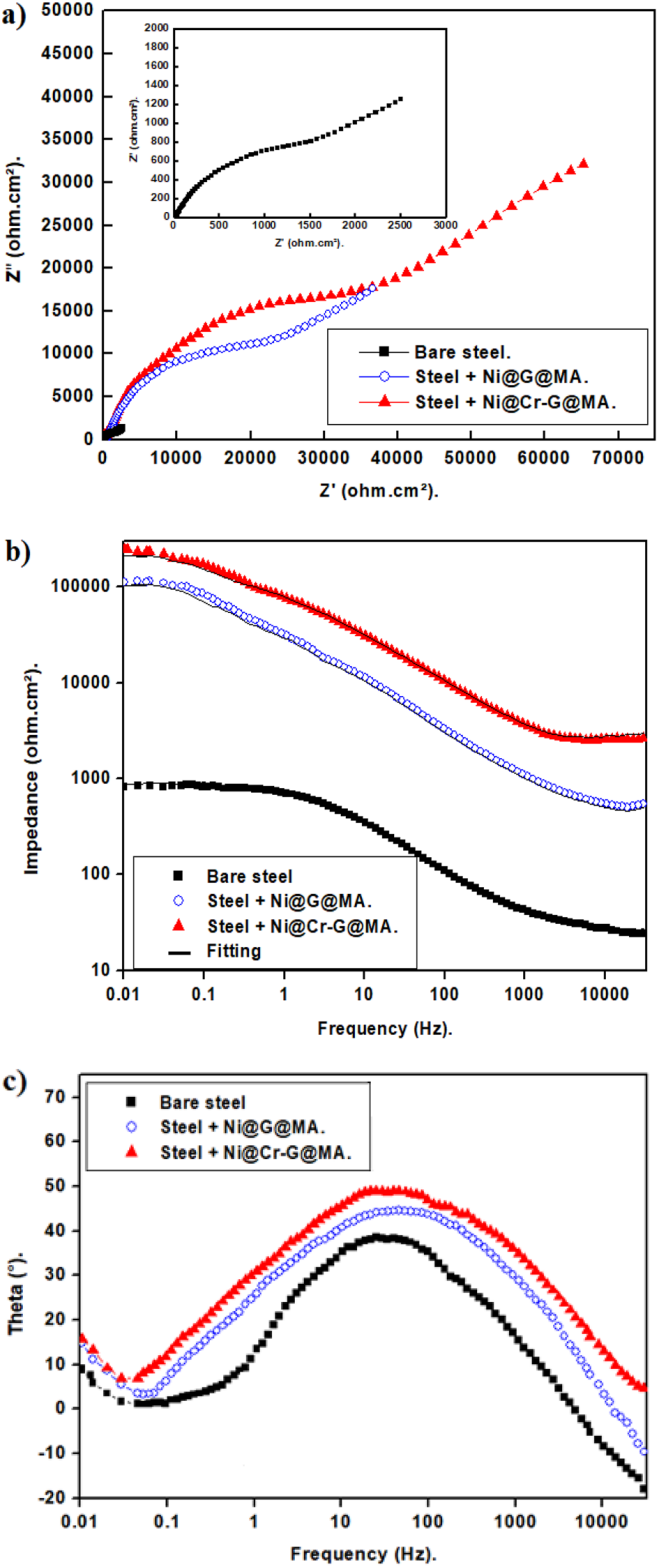
Nyquist and Bode plots of bare and SHP coated steel in 0.5 M NaCl solution.

The Bode and Nyquist diagrams of uncoated and SHP coated steel in a 0.5 M NaCl solution are displayed in Fig. 16. The Nyquist diagrams, Fig. 16a, display a depressed capacitive semicircle at a moderate frequency and a diffusion tail at a low frequency. The interfacial charge transfer reaction at moderate frequencies results in the Nyquist plots' depressed capacitive semicircle^82^. At low frequencies, the diffusion tail is caused by mass transfer from the bulk to the electrode surface. According to these findings, the steel coated with Ni@G@MA exhibits higher charge transfer resistance than bare steel because it has a protective SHP layer. The Ni@Cr-G@MA-coated steel exhibits the highest capacitive semicircle, demonstrating the highest level of protection. It is more effective for the SHP Ni@Cr-G@MA coat to reduce the diffusion of corrosive species like Cl^−^ and H_2_O into the steel metal surface as the doping of graphene with chromium enhances the SHP characteristics.

It is common to evaluate the overall corrosion performance of a protective coating using the impedance values at 0.01 Hz in Bode plots ^83^. According to Fig. 16b, produced SHP coated steel in 0.5 M NaCl solution exhibits greater impedance at 0.01 Hz on the Bode graphs compared to bare steel. This demonstrates that the developed SHP coatings protect the steel substrate. Figure 16c shows the phase angle plot; there are two times constant at low and intermediate frequencies. The time constant that developed in the low-frequency zone was caused by either the protective SHP coating or the unprotective corrosion products of bare steel. The electrical double layer was responsible for the time constant at the moderate frequency^84–86^.

**Figure 17:**
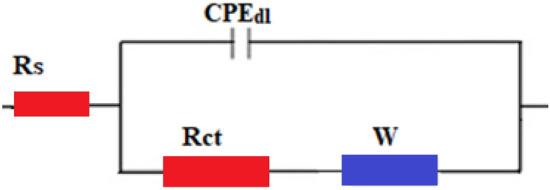
The equivalent circuit model.

The EIS experimental data were fitted utilizing the equivalent circuit shown in Fig. 17, and the impedance parameters were calculated using the Zsimpwin program. The equivalent circuit components are; solution resistance, R_s_, double-layer constant phase element, CPE_dl_, charge transfer resistance, R_ct_, and Warburg element, W. Table 3 displays the EIS parameters for bare and SHP coated steel. Equation (3) was employed to calculate the protection efficiency^80^:

3 $$\% {\text{P}} = \left[ {\left( {{\text{R}}_{{{\text{ct }} - }} {\text{R}}_{{{\text{ct}}}} ^{{\text{o}}} } \right)/{\text{R}}_{{{\text{ct}}}} } \right] \times {1}00$$

where the charge transfer resistances for the bare and SHP coated steel are R_ct_^o^ and R_ct_. It is obvious that both %P and Rct of the bare steel < steel + Ni@G@MA < steel + Ni@Cr-G@MA, and so the corrosion resistance increases in the same order. The SHP coated steel with Ni@Cr-G@MA has corrosion resistance that is higher than several previously known values^87–89^.

**Table 3:** The EIS parameters for the bare and SHP coated steel in 0.5 M NaCl solution.

Deposit	Rs (Ohm cm^2^)	n_1_	CPE_dl_ × 10^–6^ (s^n^ Ω^−1^ cm_2_)	W × 10^–4^	R_ct_ (Ohm cm^2^)	%P
Bare steel	2.3	0.75	322	511	2200	–
Steel + Ni@G@MA	4.1	0.77	33	22	36,300	93.9
Steel + Ni@Cr-G@MA	4.7	0.78	21	18	55,600	96.0

#### Mechanism of corrosion resistance performance

The bare steel can easily adsorb water molecules on its surface. Uncoated steel can also suffer severe corrosion from chloride ions adhering to its surface and forming [FeClOH]^−^^20^. On the other hand, the steel coated with SHP films has micro- and nanostructures covered in hydrophobic material. The valleys between the peaks of the rough surface are easily filled with air. Between the substrate and the corrosive environment, the air trapped on the SHP surface acts as a passivation barrier. Due to the obstructive effect of trapped air, aggressive ion species in corrosive environments, such as Cl^−^, may rarely attack the underlying surface^19,20,90^.

**Figure 18:**
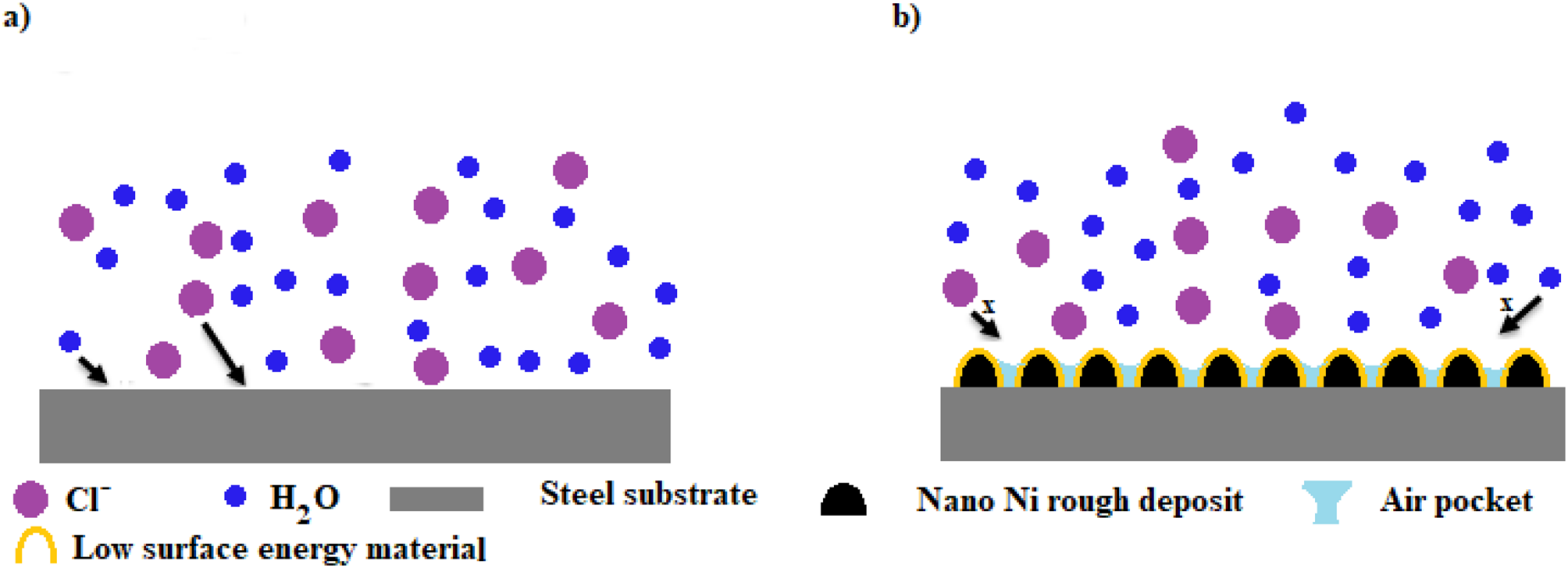
A diagram of the suggested mechanism for corrosion protection of the prepared SHP films.

Additionally, it was found that the SHP surface in neutral solutions was negatively charged since the isoelectric point for SHP materials in neutral solutions was between pH 2–4^3^. A SHP surface's negative charge led to a decline in the amount of Cl^−^ anion nearby a solid surface, which improves corrosion resistance^90^. According to reports, the existence of electronegative functional groups created at the graphite lattice gives graphene a negative zeta potential value^91,92^. So, the graphene-based SHP coating will have a very low amount of Cl^−^ anion nearby its surface, so they have a high corrosion resistance property as its high surface's negative charge. The schematic diagram of the suggested mechanism for corrosion protection of the prepared SHP coatings is shown in Fig. 18.

## Conclusion

1. We innovatively used banana leaves, an environmentally benign biomass source, to create high-quality graphene.
2. For the first time, we use the Cr-G composite in fabricating SHP coating on a steel substrate to explore its effectiveness in improving the SHP coated steel chemical stability, mechanical durability, UV durability, and corrosion resistance properties.
3. SHP Ni@G@MA and Ni@Cr-G@MA coatings were fabricated on the steel substrate with a water contact angle of 158° and 168° respectively. The SEM and AFM techniques confirm that the modification of graphene with chromium improves the prepared coat roughness, generating higher superhydrophobicity.
4. The Ni@G@MA coating maintains superhydrophobicity in the pH range of 3–11, according to the chemical stability test, whereas the Ni@Cr-G@MA coating maintains superhydrophobicity in the pH range of 1–13. The mechanical abrasion test revealed that Ni@ G@MA coating displays superhydrophobicity up to an abrasion length of 1200 mm while SHP Ni@ Cr-G@MA coating displays superhydrophobicity up to an abrasion length of 2500 mm. The Ni@G@MA film maintains superhydrophobicity until 10 cycles of the sand impact, while the Ni@Cr-G@MA film maintains its superhydrophobicity up to 16 cyclic of the sand impact.
5. The SHP coated steel by Ni@G@MA has UV stability for 60 h, while the coated steel by Ni@Cr-G@MA has UV stability up to 110 h.
6. The PDP and EIS results show that the coating of steel with a SHP coating greatly increases the protection efficiency, especially if the graphene-based coat is doped with chromium. So, doping the SHP Ni@G@MA coating with chromium giving Ni@Cr-G@MA greatly improves the coated steel's chemical, mechanical, UV durability, and corrosion resistance behaviour.

## Data Availability

The datasets used and/or analyzed during the current study are available from the corresponding author upon reasonable request.
